# Antibacterial Effects of Magnetically-Controlled Ag/Fe_3_O_4_ Nanoparticles

**DOI:** 10.3390/ma11050659

**Published:** 2018-04-24

**Authors:** Ming Chang, Wei-Siou Lin, Weihao Xiao, Yi-Ning Chen

**Affiliations:** 1 Key Laboratory of Process Monitoring and System Optimization for Mechanical and Electrical Equipment in Fujian Province, Huaqiao University, Xiamen 361021, China; ming@cycu.edu.tw; 2 Department of Mechanical Engineering, Chung Yuan Christian University, Chung Li 32023, Taiwan; hugh0322452@hotmail.com (W.-S.L.); sacre@gmail.com (W.X.); 3 Department of Bioscience Technology, Chung Yuan Christian University, Chung Li 32023, Taiwan

**Keywords:** Ag/Fe_3_O_4_ nanoparticles, rotating magnetic field, *Escherichia coli*, antibacterial effect

## Abstract

This paper presents the use of a magnetic manipulation device to remotely control the movement of Ag/Fe_3_O_4_ nanoparticles (NPs) for enhancing the antibacterial effect of Ag particles in aqueous suspensions containing *Escherichia coli* (*E.* *coli*). The Ag/Fe_3_O_4_ magnetic NPs were prepared by co-precipitation method where the Ag particles are simultaneously synthesized with the Fe_3_O_4_ particles to form Ag and Fe_3_O_4_ nanocomposite materials. The manipulation system utilized a homogeneous rotating magnetic field to carry out magnetic stirring of NPs in the petri dishes containing bacterial suspensions. The optimum magnetron parameters and best antibacterial effects were implemented with six different concentrations from 0.6 wt % to 6.6 wt % of the NPs at driving frequencies from 50 rpm to 200 rpm for 3 min. The highest antibacterial effect of 99.4% was achieved at 5.4 wt % of NPs and the driving frequency of 100 rpm. A time-dependent antibacterial effect in 0.1 wt % of Ag/Fe_3_O_4_ was also observed. The results indicate that the use of specific rotating magnetic fields to manipulate Ag/Fe_3_O_4_ magnetic NPs can significantly improve the antibacterial efficacy. Due to the good biocompatibility of the Ag NPs, the presented technique can be applied to clean water resources in the future.

## 1. Introduction

Silver has been utilized as a bacteriostatic material for ages because Ag ions can kill bacteria by punching holes in bacterial membranes and damaging them inside. Apart from its excellent antibacterial effect, harmful byproducts are not generated during the antibacterial process of Ag sterilization like chloride derives. Hwang et al. [1] tested the efficacy of Ag ions against *Legionella pneumophila*, *Pseudomonas aeruginosa* and *Escherichia coli* (*E. coli*) in drinking water. Silvestry-Rodriguez et al. [2] launched a study in using Ag to reduce the activity of *Pseudomonas aeruginosa* and *Aeromonas hydrophila* in tap water and to evaluate the possibility for using Ag to replace or reduce the dosage of chlorine, harmful for human health and environment. Zhang [3] compared various techniques for using Ag nanoparticles (NPs) in purifying drinking water. In general, the antimicrobial efficacy of Ag ions can be enhanced by increasing the content of Ag particles. The extensive application of the Ag particles results in their inevitable release into the environment [1,2,3]. If Ag particles can be manipulated in some efficient way to increase their contact surface or frequencies with bacteria, the higher antibacterial effect could be expected with the lower concentration of Ag particles and the risks of environmental contamination from Ag particles can be reduced.

Over the past decades, magnetic NPs were widely applied to magnetic targeting medical tools [4,5], magnetic resonance imaging agents [6,7] and the carriers of the Ag-loading antimicrobials [8,9]. Among the various magnetic support materials, Fe_3_O_4_ nanostructure has gained much attention in the fields of biomedical and environmental engineering due to their stable magnetic properties and favorable biocompatibility [10,11,12]. Silver-coated Fe_3_O_4_ NPs as functional antimicrobial agents has now become an important research topic [13,14]. In the current study, a portable magnetic manipulation device was developed to remotely control the movement of Ag/Fe_3_O_4_ NPs to increase the contact of Ag ions with *E. coli* for enhancing the antibacterial effects of Ag particles by stirring the magnetic NPs in petri dishes containing *E. coli*. The use of a magnetic field is extremely versatile because the field strength and its orientation can be controlled. The rotating magnetic fields applied to induce a magnetic torque can drive the stirring action of the Ag/Fe_3_O_4_ NPs. The antibacterial effect of the Ag/Fe_3_O_4_ NPs on *E. coli* was investigated at different rotation speeds and different concentrations of the NPs in the suspensions. The results showed that the antibacterial effect of Ag in these aqueous suspensions was greatly improved by magnetically stirring the Ag/Fe_3_O_4_ NPs in the magnetic manipulation system. Therefore, utilizing magnetically-controlled Ag/Fe_3_O_4_ NPs in aqueous suspensions can be applied to water purification and other sterilization-related objectives. 

## 2. Materials and Methods 

### 2.1. Experimental System and Mechanism

**Figure 1:**
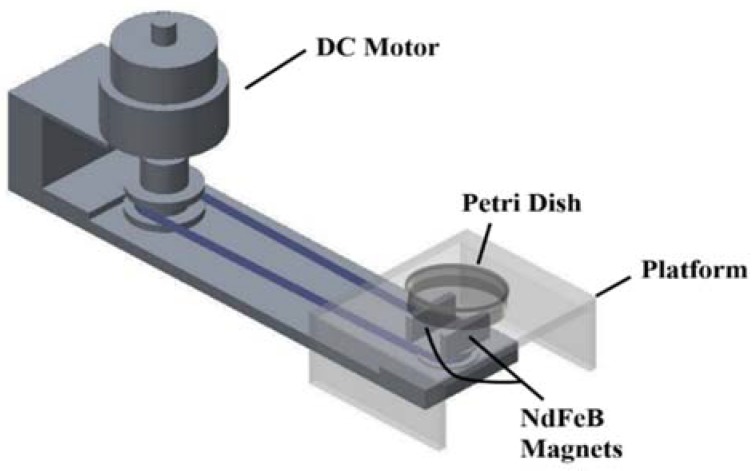
Schematic of the magnetic manipulation device.

The major components of the experimental system included a magnetic manipulation device to manipulate the motion of Ag/Fe_3_O_4_ NPs in the petri dishes containing bacterial suspensions. Figure 1 shows the magnetic manipulation device that was implemented to generate a rotating homogeneous magnetic field. The rotation of magnetic NPs was subjected to the competing torques generated by the external magnetic field and viscous drag force of the suspension. The rotating magnetic field system was constructed using two NdFeB permanent magnets (~6.6 mT). The magnets were mounted on a rotary-motorized stage where the motor speed was tunable from 50 rpm to 200 rpm. The antibacterial effects of the NPs were investigated for different concentrations and rotation speeds of the NPs in the suspension.

The rotation of the NPs was produced by the interaction of the induced magnetization to the oscillating magnetic field. The magnitude of the magnetic torque needed to rotate the NPs can be calculated by Equation (1) where *V* is the volume of the NPs, χ is the material susceptibility, μ_0_ is the vacuum permeability, *H* is the magnetic field strength, and θ is the angle between the magnetic dipole moment of the NPs and *H* [15].

(1) $$\tau_{m}=V\frac{\chi^{2}}{2\left(2+\chi \right)}\mu_{0}H^{2}\sin\left(2\theta \right)$$

For a NP of radius *r* in a fluid with viscosity η, the rotation is subjected to a viscous drag force, $\tau_{drag}=3\pi^{2}\omega \eta r^{4}$, which counterbalances the magnetic torque. Therefore, the final rotation speed of the NPs can be calculated using Equation (2) suggesting that the NPs can be rotated in a controllable fashion by modulating the time-dependent magnetic field source. Thus, the stirring of the NPs can be stopped or resumed by simply switching the DC source.

(2) $$\omega =\frac{Vx^{2}\mu_{0}H^{2}\sin\left(2\theta \right)}{3\pi^{2}\eta r^{4}}$$

### 2.2. Preparation of Ag/Fe_3_O_4_ NPs

In this study, Ag/Fe_3_O_4_ NPs were prepared by the co-precipitation reaction of Fe(II) and Fe(III) with ammonia hydroxide. After 16.98 g of silver nitrate and 27.8 g of ferrous sulfate were dissolved in 100 mL of deionized water as the precursor solutions, the solutions were uniformly mixed and ammonia hydroxide was added using a stirrer to make sure the precursors were dissolved in a basic solution (pH ≥ 9). Silver ions were used as oxidation agent to oxidize ions of Fe(II) to form a solution containing both Fe(II) and Fe(III) ions. Chemical reduction and oxidation were utilized as Equation (3):

Fe^2+^ + Ag^+^ → Fe^3+^ + Ag^0^ (3)

and Equation (4):

Fe^3+^ + Fe^2+^ + 8OH^−^ → Fe_3_O_4_ + 4H_2_O. (4)

Through precipitation with ammonia hydroxide, magnetic NPs containing Ag metals were obtained. The precipitates were processed next by a microwave hydrothermal process at 150 °C for 30 min. Finally, the materials were washed with deionized water until the pH value reached 7 and then the neutral materials were oven-dried at 70 °C for 24 h. The dried Ag/Fe_3_O_4_ NPs were then ground into powder using an agate mortar. In addition, bare Fe_3_O_4_ NPs were also prepared using a similar procedure without silver nitrate precursor [16].

### 2.3. Characterization of Ag/Fe_3_O_4_ NPs

**Figure 2:**
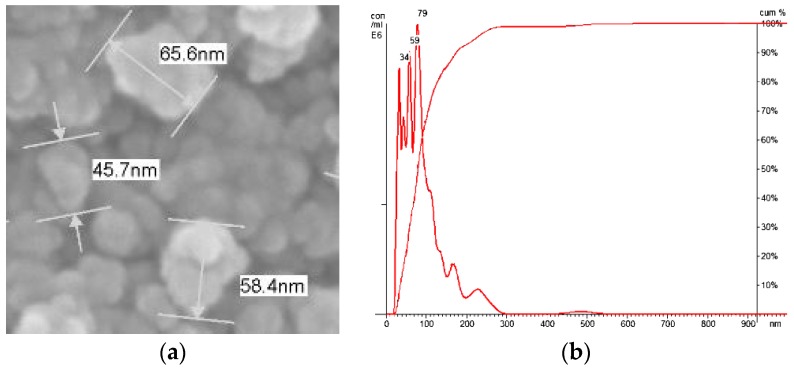
(**a**) Scanning electron microscopy (SEM) image of Ag/Fe_3_O_4_ nanoparticles (NPs); and (**b**) size distribution of Ag/Fe_3_O_4_ NPs between 34 nm and 79 nm by nanoparticle tracking analysis and NanoSight LM100.

Morphology of the prepared Ag/Fe_3_O_4_ NPs is shown in Figure 2. Figure 2a is a scanning electron microscopy (SEM) image of the Ag/Fe_3_O_4_ NPs, showing the diameters of these NPs ranged from 30 nm to 80 nm. The agglomeration of Ag/Fe_3_O_4_ NPs can be observed. The size distribution of the prepared Ag/Fe_3_O_4_ NPs was measured by NanoSight LM100 (Malvern Panalytical, Malvern, UK) and analyzed by nanoparticle tracking analysis (NTA) software according to the light scattering and Brown motion of the materials in the suspension. Figure 2b is the size distribution of Ag/Fe_3_O_4_ NPs between 34 nm and 79 nm. The measurements in the SEM image were consistent with those measured by NanoSight LM100.

**Figure 3:**
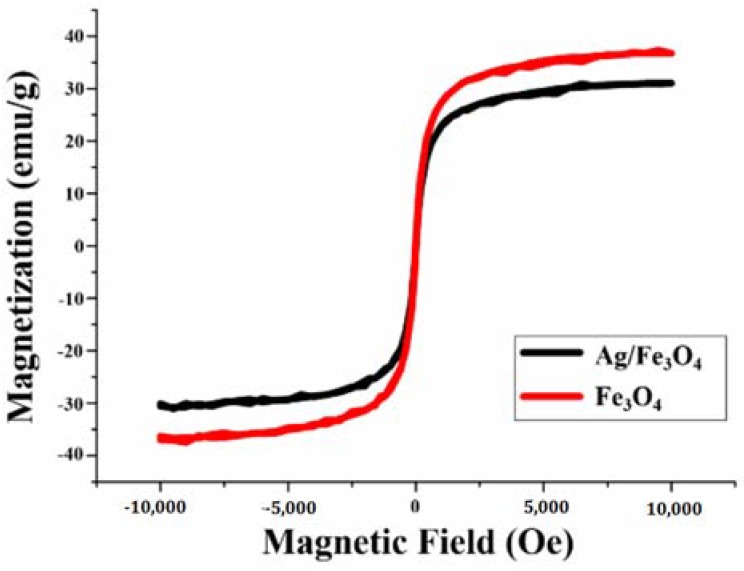
Magnetic saturation of bare Fe_3_O_4_ and Ag/Fe_3_O_4_ nanoparticles.

The magnetizations of bare Fe_3_O_4_ and Ag/Fe_3_O_4_ NPs were measured using vibrating sample magnetometer (VSM), as shown in Figure 3. There was almost no hysteresis when the magnetic field ranged from −10,000 to 10,000 Oe. The saturation magnetizations of bare Fe_3_O_4_ and Ag/Fe_3_O_4_ NPs were 40.4 emu/g and 31.1 emu/g, respectively. The results confirmed the good magnetic characteristics of the prepared NPs.

**Figure 4:**
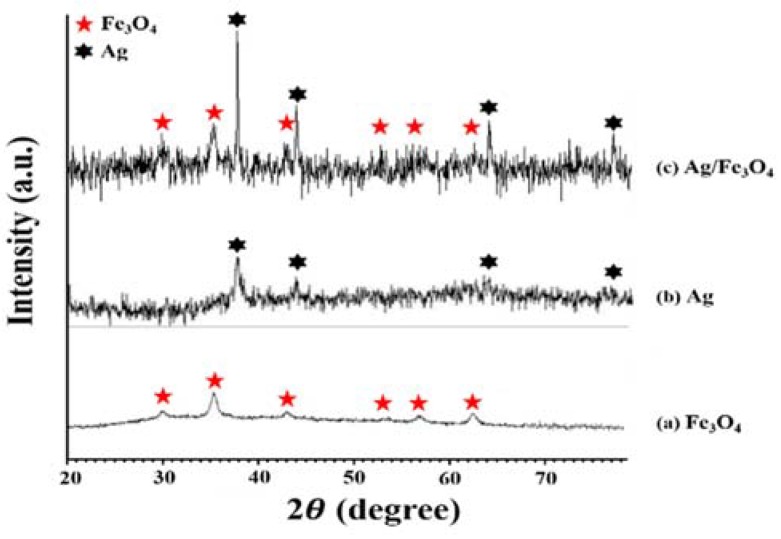
X-ray diffractometer (XRD) patters of the prepared nanoparticles: (**a**) Fe_3_O_4_; (**b**) Ag; and (**c**) Ag/Fe_3_O_4_.

The crystallization behavior of the synthesized NPs was analyzed by an X-ray diffractometer (XRD). As shown in Figure 4, both the peaks at (220), (311), (400), (422), (511), and (440) with respective crystal planes at 2θ = 30.1°, 35.4°, 43.1°, 53.4°, 56.9°, and 62.5° on Fe_3_O_4_ and (111), (200), (220), and (311) with respective crystal planes at 2θ = 38.1°, 44.2°, 64.4°, and 77.5° on Ag are clearly displayed. The XRD patterns reveal that the crystallinity of the NPs agrees with the standard values of Ag/Fe_3_O_4_ composite materials.

### 2.4. Antibacterial Effects of Ag/Fe_3_O_4_ NPs

Experiments were carried out to investigate the antibacterial effects of magnetically controlled Ag/Fe_3_O_4_ NPs on *E. coli*. *E. coli* (ATCC 10,322, Biosafety level 1) was selected and cultured in lysogeny broth (LB) media at 37 °C for 16 h for the antibacterial tests. LB broth was prepared by dissolving 2.5 g of yeast extract, 5 g of tryptone, and 5 g of NaCl into 487.5 mL of deionized water and then autoclaving at 121 °C for 45 min. The first experiment was executed to find out the optimum operation mechanism. The original content of *E. coli* in petri dishes was quantified according to the absorption intensity of the bacterial suspensions using a spectrophotometer at the wavelength of 600 nm (OD_600nm_) [17]. The aliquots from the same *E. coli* suspension were used to test six concentrations of Ag/Fe_3_O_4_ NPs, i.e. 0.6 wt %, 1.8 wt %, 3.0 wt %, 4.2 wt %, 5.4 wt %, and 6.6 wt %, to explore the highest antibacterial effect and the optimum magnetron parameters. The suspensions of Ag/Fe_3_O_4_ NPs were stirred by utilizing the motor in the magnetic manipulation device with driving frequencies of 50 rpm, 80 rpm, 100 rpm, 120 rpm, and 200 rpm, for 3 min per test. After the magnetic stirring, the Ag/Fe_3_O_4_ NPs in the suspensions were attracted to the bottom of the petri dish via a magnet and the supernatants were taken out to check the amount of the remaining *E. coli* by measuring OD_600nm_. The residual bacteria rates (%) for the tests on different rotation speeds and NP weight concentrations were calculated using Equation (5) where N_origin_ was the OD_600nm_ of the *E. coli* suspension without NPs before tests and N_sample_ was the OD_600nm_ of each supernatant under different conditions after magnetic stirring.

Residual bacteria rate (%) = N_sample_/N_origin_ × 100% (5)

### 2.5. Statistical Analysis

Residual bacteria rates obtained from different treatments were analyzed by the ANOVA with the significant *p*-value (<0.05) by using Excel software.

## 3. Results and Discussion

**Figure 5:**
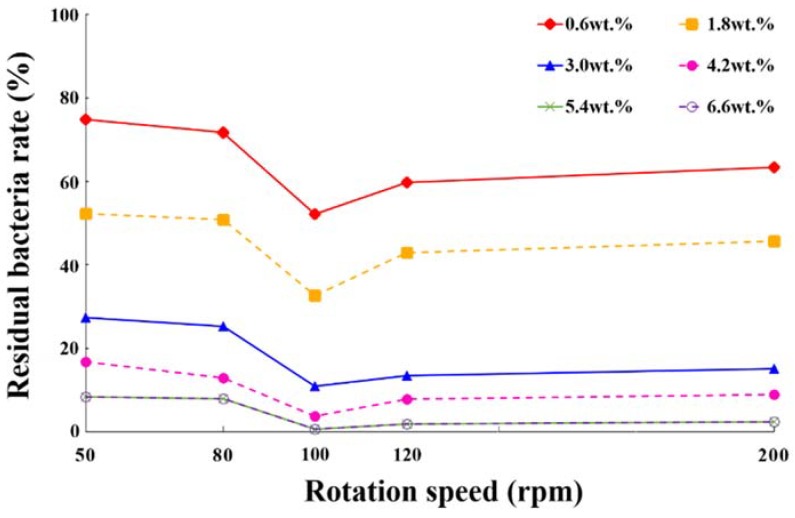
Residual bacterial rate under different Ag/Fe_3_O_4_ concentrations and motor driving frequencies.

The experiment investigating the antibacterial effects of magnetically controlled Ag/Fe_3_O_4_ NPs under different rotation speed and NP weight concentrations showed that the lowest residual bacterial rate (0.6%) was achieved at the concentration of Ag/Fe_3_O_4_ NPs at 5.4 wt % and 6.6 wt % with driving frequency of 100 rpm after 3 min (Figure 5). Because the concentration of 5.4 wt % and 6.6 wt % showed the same residual bacterial rates with five different driving frequencies, the NPs in the solution have been saturated at the concentration of 5.4 wt % and the highest antibacterial effect reached 99.4% with driving frequency of 100 rpm. This may be because higher movement speed of the NPs reduces the contact time between particles and bacteria and results in the bacteriostatic efficiency decreasing slightly for a higher driving frequency.

**Figure 6:**
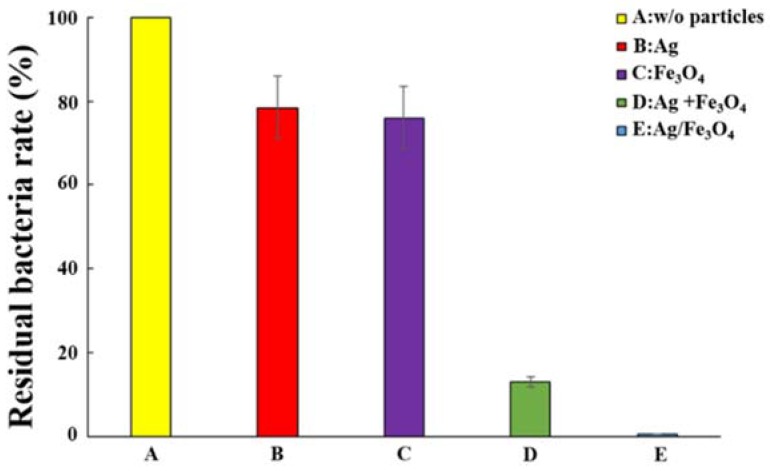
Comparison of antibacterial effects with different kinds of nanoparticles (NPs): (**A**) bacterial solution only; (**B**) solution with only non-magnetic Ag NPs; (**C**) solution with only Fe_3_O_4_; (**D**) solution with both Ag NPs and Fe_3_O_4_ NPs; and (**E**) solution with Ag/Fe_3_O_4_ NPs.

The antibacterial effects obtained from pure Ag and/or Fe_3_O_4_ NPs were also investigated for comparisons. Under the same NP concentration of 5.4 wt % and driving frequency of 100 rpm, the residual bacterial rates of the following were compared: (A) bacterial suspension only; (B) suspension with only non-magnetic Ag NPs; (C) suspension with only Fe_3_O_4_; (D) suspension with both Ag NPs and Fe_3_O_4_ NPs; and (E) suspension with Ag/Fe_3_O_4_ NPs (each magnetically stirred 3 min) (Figure 6). The lowest residual bacterial rate indicating the strongest antibacterial effect was observed in the suspension of Ag/Fe_3_O_4_ NPs (E), followed by the suspension with both Ag NPs and Fe_3_O_4_ NPs (D). The suspension with only non-magnetic Ag NPs (B) exhibited the worst antibacterial effect, similar to that of the suspension with only Fe_3_O_4_ NPs without Ag (C). Statistically significant differences (*p*-value < 0.05) were found between every pair of groups except Group Ag (B) and Group Fe_3_O_4_ (C) among Groups B–E after ANOVA analysis by Excel software. The agitation of Fe_3_O_4_ NPs can push the movement of Ag NPs to increase the contact between Ag particles and bacteria, which reduced the residual bacterial rate of solution with only Ag NPs (B) from 80% to 18% from the solution with both Ag NPs and Fe_3_O_4_ NPs. The results indicated that manipulating Ag/Fe_3_O_4_ magnetic NPs in the solutions can substantially increase the antibacterial ability of Ag NPs. 

**Figure 7:**
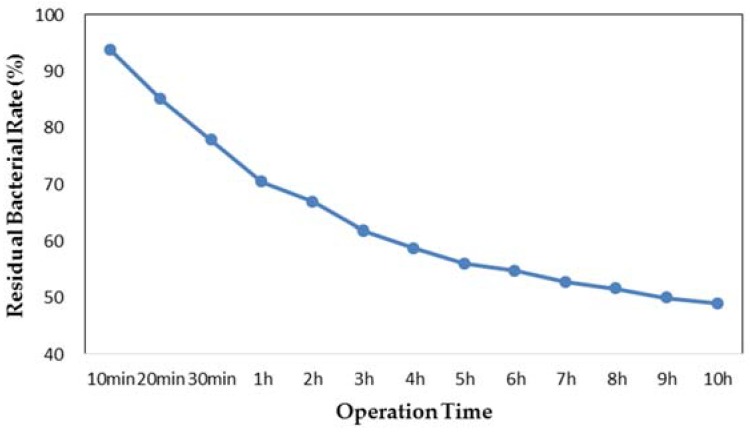
Antibacterial effect to *Escherichia coli* from 0.1 wt % of Ag/Fe_3_O_4_ with driving frequency of 100 rpm.

To investigate the sterilization performance of low concentration of Ag/Fe_3_O_4_ NPs, a bacterial suspension with 0.1 wt % concentration of the NPs was prepared and manipulated at the driving frequency of 100 rpm. The time-dependent antibacterial effect observed from the suspension is shown in Figure 7. The bacteria were quickly eliminated from 94% to 70% in the first hour. The trend then eased gradually after 3 h of treatment. The final residual bacterial rate was roughly 48%. This may be because the Ag/Fe_3_O_4_ NPs is low in content and the dead bacterial would attach to the surface of NPs, causing the contact between NPs and bacteria to gradually decrease.

## 4. Conclusions

In this study, magnetic Ag/Fe_3_O_4_ NPs were synthesized with co-precipitation method and added into *E. coli* suspensions to explore the application potential of water purifying platform based on the nanocomposite materials. The magnetic NPs were stirred with a custom-made magnetic manipulation system. Combined with the antimicrobial properties of Ag and the magnetically controlled Fe_3_O_4_ NPs, the antibacterial effects on *E. coli* at different concentrations and rotation speeds of the magnetic Ag/Fe_3_O_4_ NPs were investigated. Experimental results showed that magnetic manipulation can significantly improve the contact of Ag NPs and bacteria and thus the antibacterial abilities of Ag NPs. The strongest antibacterial effect was obtained at the concentration of 5.4 wt % of Ag/Fe_3_O_4_ NPs with driving frequency of 100 rpm, where there was only 0.6% of the original bacterial concentration left. This shows that the *E. coli* in suspensions can be almost completely eradicated in a short time with the proposed method. Since these magnetic nanomaterials can be recycled and reused, the magnetically-controlled Ag/Fe_3_O_4_ NPs would be a cost-effective and efficient technique for water purification and other sterilization-related applications.
